# Mitofusin‐2 knockdown increases ER–mitochondria contact and decreases amyloid β‐peptide production

**DOI:** 10.1111/jcmm.12863

**Published:** 2016-05-20

**Authors:** Nuno Santos Leal, Bernadette Schreiner, Catarina Moreira Pinho, Riccardo Filadi, Birgitta Wiehager, Helena Karlström, Paola Pizzo, Maria Ankarcrona

**Affiliations:** ^1^ Center for Alzheimer ResearchDivision of NeurogeriatricsDepartment of Neurobiology, Care Sciences and SocietyKarolinska InstitutetStockholmSweden; ^2^ Department of Biomedical SciencesUniversity of PaduaPaduaItaly

**Keywords:** Alzheimer's disease, Mitofusin‐2, ER–mitochondria contacts, Aβ, γ‐secretase

## Abstract

Mitochondria are physically and biochemically in contact with other organelles including the endoplasmic reticulum (ER). Such contacts are formed between mitochondria‐associated ER membranes (MAM), specialized subregions of ER, and the outer mitochondrial membrane (OMM). We have previously shown increased expression of MAM‐associated proteins and enhanced ER to mitochondria Ca^2+^ transfer from ER to mitochondria in Alzheimer's disease (AD) and amyloid β‐peptide (Aβ)‐related neuronal models. Here, we report that siRNA knockdown of mitofusin‐2 (Mfn2), a protein that is involved in the tethering of ER and mitochondria, leads to increased contact between the two organelles. Cells depleted in Mfn2 showed increased Ca^2+^ transfer from ER to mitchondria and longer stretches of ER forming contacts with OMM. Interestingly, increased contact resulted in decreased concentrations of intra‐ and extracellular Aβ_40_ and Aβ_42_. Analysis of γ‐secretase protein expression, maturation and activity revealed that the low Aβ concentrations were a result of impaired γ‐secretase complex function. Amyloid‐β precursor protein (APP), β‐site APP‐cleaving enzyme 1 and neprilysin expression as well as neprilysin activity were not affected by Mfn2 siRNA treatment. In summary, our data shows that modulation of ER–mitochondria contact affects γ‐secretase activity and Aβ generation. Increased ER–mitochondria contact results in lower γ‐secretase activity suggesting a new mechanism by which Aβ generation can be controlled.

## Introduction

Organelles are dynamic and in contact with each other. One example is the endoplasmic reticulum (ER) that is physically and biochemically connected with the outer membrane of mitochondria (OMM). These connections occur at subregions of the ER membrane referred to as mitochondria‐associated ER membranes (MAM). Mitochondria‐associated ER membranes are specialized cholesterol‐rich stretches of ER membranes with similar properties and composition as lipid rafts 1. The distance between ER and OMM at the contacts is approximately 20–30 nm 2. Several proteins have been described to tether ER and mitochondria in mammalian cells. Such proteins include: mitofusin‐2 (Mfn2), phosphofurin acidic cluster sorting protein‐2, inositol 1,4,5‐triphosphate receptor 3 (IP3R3), glucose‐regulated protein and voltage‐dependent anion channel 1 (VDAC1) 3, 4, 5, 6. Mitofusin‐2 is located both in MAM and OMM, while Mfn1 is restricted to the OMM 6. Previous studies have suggested that mitofusin homo‐ and heterodimers (Mfn2–Mfn2 and Mfn2–Mfn1) form an ER–mitochondria tethering complex. However, recent data propose that Mfn2 instead acts as a negative regulator, preventing excess of contacts between the two organelles 7, 8, 9. These studies show that reduction of Mfn2 in mouse embryonic fibroblasts results in increased ER–mitochondria coupling and Ca^2+^ transfer 7, 8. Ca^2+^ is released at MAM *via* the IP3Rs and taken up by mitochondria *via* VDAC1 (in the OMM) and the mitochondrial Ca^2+^ uniporter (MCU, in the inner mitochondrial membrane) 3, 10, 11, 12. Moreover, it has been shown that ER–mitochondria tethering and the Ca^2+^ transfer between the two organelles are enhanced in cells expressing a familial Alzheimer's disease mutation in presenilin‐2 (PS2) 13 .

Other functions of MAM and ER–mitochondria communication include: phospholipid and cholesterol synthesis and trafficking, formation of autophagosomal membranes and regulation of apoptosis 14, 15, 16. Interestingly, all these processes are affected in Alzheimer's disease (AD) pathogenesis 1, 10, 17, 18, 19, 20, 21, 22, 23. We have previously reported that the expression of MAM‐associated proteins is up‐regulated in AD *postmortem* tissues and that primary neurons exposed to Aβ show an increased number of ER–mitochondria contacts as detected by the proximity ligation assay 24. Other studies show that the synthesis of cholesteryl esters and phospholipids is increased in fibroblasts derived from AD patients and in cells treated with apolipoprotein ε4‐conditioned medium 14, 25.

Alzheimer's disease is a multifactorial neurodegenerative disease characterized by several neurological impairments. Pathological hallmarks include accumulation of extracellular amyloid plaques and intraneuronal fibrillary tangles 26, 27, 28. The amyloid β‐peptide (Aβ) is the main component of amyloid plaques. Aβ is generated *via* proteolytic processing of the amyloid‐β precursor protein (APP) by two enzymes: β‐site APP‐cleaving enzyme 1 (BACE1) and the γ‐secretase complex. The γ‐secretase complex consists of four different proteins: Nicastrin (NCT), presenilin enhancer 2 (PEN‐2), anterior pharynx‐defective 1 (APH‐1) and PS1 or PS2 29, 30, 31, 32. In the amyloidogenic pathway, APP is first cleaved by BACE1 generating sAPPβ and C99. C99 is subsequently cleaved by γ‐secretase to generate Aβ and APP intracellular domain (AICD) 29, 30, 31, 32. In the non‐amyloidogenic pathway APP is first cleaved by α‐secretase which generates sAPPα and C83. The C83 fragment is subsequently cleaved by γ‐secretase generating a p3 fragment and AICD.

Several studies have shown enrichment of APP, PS1/PS2, Aβ as well as γ‐secretase activity in lipid rafts and MAM 14, 33, 34. Accordingly, we recently demonstrated that significant amounts of Aβ_40_ and Aβ_42_ are generated from MAM‐enriched subcellular fractions of mouse brain 34. Thus, a fraction of Aβ is generated in the vicinity of mitochondria, where it could exert a toxic effect.

Here, we have investigated the role of ER–mitochondria interplay in the regulation of Aβ production. Our data show that siRNA knockdown of Mfn2 results in increased contact between the two organelles leading to increased Ca^2+^ transfer from ER to mitochondria and decreased Aβ concentrations. Interestingly, γ‐secretase complex maturation and activity is impaired in these conditions revealing a new mechanism by which cells regulate Aβ production.

## Material and methods

Additional details are given in Data S1.

### Cell viability and ATP levels

Cell viability was measured using the dye alamarBlue^®^ (#DAL1025; Thermo Fisher Scientific, Waltham, MA, USA) and Mitochondrial ToxGlo^™^ (#G800; Promega , Madison, WI, USA). In the first assay, cells were incubated with 1× AlamarBlue^®^ solution for 30 min. at 37°C, and part of the sample media was removed and fluorescence (λ_ex_ = 530–560 nm, λ_em_ = 580–610 nm) was measured. With Mitochondrial ToxGlo^™^ both cell viability and ATP levels were measured according to the manufacturer's protocol.

### Transmission electron microscopy

Cells were pelleted and fixed in 2.5% (vol/vol) glutaraldehyde in 0.1 M phosphate buffer at room temperature, rinsed in 0.1 M phosphate buffer, post‐fixed in 2% OsO_4_ for 2 hrs, dehydrated in ethanol and acetone and finally embedded in LX‐112 (Ladd, Burlington, VT, USA). Ultrathin sections were prepared using Leica Ultracut UCT (Leica, Wien, Austria) and contrasted with uranyl acetate followed by lead citrate. Specimens were examined in a Tecnai 12 BioTWIN transmission electron microscope (FEI Company, Eindhoven, The Netherlands) at 100 kV. Digital images were taken with a Veleta camera (Olympus Soft Imaging Solutions, GmbH, Münster, Germany) at a primary magnification of 20,500×. Pictures were then analysed using Image J software (National Institutes of Health, Bethesda, MD, USA).

### Aequorin Ca^2+^ measurements

Aequorin measurements were performed as previously described 13 and are summarized in Data S1.

### Determination of intra‐ and extracellular Aβ_40_ and Aβ_42_ concentrations

Twenty‐four hours after the initiation of siRNA treatment cell medium was replaced by OptiMEM. After another 24 hrs, the OptiMEM was collected for quantification of extracellular Aβ levels. At the same time cells were lysed and intracellular Aβ levels measured. About 10 μg of protein from each sample (lysed cells or conditioned medium) was mixed with RIPA buffer and loaded in duplicates on the ELISA plate. The amounts of Aβ_40_ and Aβ_42_ were measured using the Human/rat Aβ_40_ (#294‐62501) or Aβ_42_ (#290‐62601) ELISA Kit Wako (Wako Chemicals, GmbH, Neuss, Germany) according to the manufacturer's instructions.

### γ‐secretase activity assay

All procedures were performed on ice or at 4°C. First, cells were harvested and spun down at 1500 × g, for 5 min. The supernatant was removed and pellet resuspended in buffer H [20 mM HEPES, 150 mM NaCl, 2 mM ethylenediaminetetraacetic acid (EDTA)]. Cells were then lysed by using G27 needles (Misawa) (130 strokes) and centrifuged at 1000 × g for 15 min. The pellet (nuclei fraction) was discarded while the supernatant (membrane fraction) was centrifuged at 100,000 × g for 1 hr. Pellet was then solubilized in buffer H containing 1% CHAPSO. Samples were shaken for 1 hr, and centrifuged again at 10,000 × g for 5 min. to remove insolubilized membranes. The supernatant was then diluted 1:2 with buffer H and 20 μg of protein incubated either with DMSO (control) or L‐685,458 for 16 hrs at 37°C. Subsequently, sample buffer was added and Western blot analysis performed.

### Neprilysin activity assay

Cells, grown in black 96‐well plates with a clear bottom, were washed with 0.1 M MES (pH = 6.5) and incubated with substrate mix (0.1 M MES (pH = 6.5), 1× complete protease inhibitor (#786‐331; G‐Biosciences, Maryland Heights, MO, USA), 1 μM Z‐Leu‐Leu‐Leu‐H (aldehyde) (#3175; Peptide Institute, Osaka, Japan), 0.5 mM Suc‐Ala‐Ala‐Phe‐MCA (#S8758; Sigma‐Aldrich, St. Louis, MO, USA) with or without 10 μM Thiorphan (#T6031; Sigma‐Aldrich) for 1 hr at 37°C. The cells were then incubated with 15 μM Phosphoramidon (#4082; Peptide Institute) and 0.14 μl of LAPase (#L5006; Sigma‐Aldrich) in a final volume of 100 μl. Reaction was stopped with 10 nM of EDTA and fluorescence was measured in a plate reader (λ_Ex_ = 355 nm, λ_Em_ = 460 nm). Differences between substrate mix with and without thiorphan represent neprilysin activity.

### Statistical analysis

All data were analysed using IBM SPSS Statistics 22 software (IMB Corporation, New York, NY, USA). Data were evaluated for normal distribution using Kolmogorov–Sminorv test, checking the skewness of the distribution and Q–Q plot. Normally distributed data were compared by two‐tailed independent‐samples *t*‐test. Not normally distributed data were compared by non‐parametric, independent Mann–Whitney *U*‐test. All values are expressed as mean ± S.E.M., *n* corresponds to number of independent experiments, *P < 0.05 and **P < 0.001 were considered to be significant.

## Results

### Cell viability and ATP levels are maintained during Mfn2 knockdown

**Figure 1:**
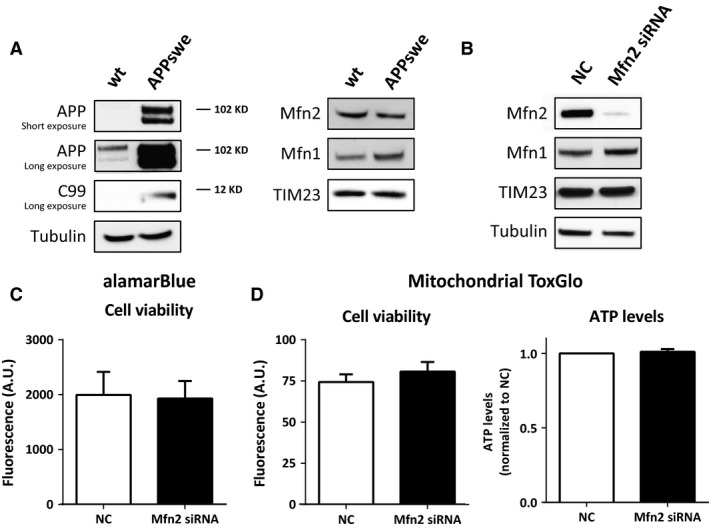
Mfn2 siRNA knockdown in HEK293 cells stably overexpressing APPswe does not affect cell viability and ATP levels. (**A**) Whole cell homogenate of HEK293 WT and APPswe cells were subjected to SDS‐PAGE and Western blot. Membranes were stained for indicated proteins. (**B**) Western blot of crude homogenate from HEK293 APPswe cells treated with NC or Mfn2 siRNA for 48 hrs. Membranes were stained for the indicated proteins. TIM23 was used as mitochondrial marker. (**C**) Cell viability (cytosolic reducing power) was measured using alamarBlue assay and presented in fluorescence arbitrary units (A.U.). (**D**) Cell viability measured as cell membrane permeability (fluorescence A.U.) and ATP levels (luminescence normalized to NC) were detected using Mitochondrial ToxGlo assay. Results are shown in mean ± S.E.M. of four independent experiments (*n* = 4) and duplicates for each condition. Mann–Whitney *U*‐test was used for statistical analysis. NC: negative control; Mfn2 siRNA: siRNA for Mfn2 mRNA.

HEK293 cells stably overexpressing APP Swedish mutation (HEK293 APPswe) were used as a model system. The Swedish double mutation (KM670/671NL), located close to the BACE1 cleavage site on APP, enhances generation of the γ‐secretase substrate C99 and consequently Aβ 35, 36, 37, 38. Overexpression of APPswe in HEK293 cells was confirmed by Western blot (Fig. 1A). The expression of Mfn2 and mitochondrial marker translocase of the inner membrane subunit 23 (TIM23) was not affected by the APPswe overexpression (Fig. 1A). To modulate ER–mitochondria contacts, cells were treated with Mfn2 siRNA or negative control (NC) siRNA for 48 hrs (Fig. 1B). Down‐regulation of Mfn2 was accompanied by a slight increase in Mfn1 expression, while TIM23 expression was not affected (Fig. 1B).

The effect of Mfn2 knockdown on cell viability and energy production was investigated using the alamarBlue^®^ and Mitochondrial ToxGlo^™^ assays. No differences in cell viability (reducing power and plasma membrane permeability) and ATP levels were detected between NC‐ and Mfn2 siRNA‐treated cells (Fig. 1C and D). Analysis of cell morphology in a bright field microscope revealed that cells maintained their morphology during the treatment with Mfn2 siRNA (data not shown).

### Knockdown of Mfn2 leads to extended ER–mitochondria contact length and increased Ca^2+^ transfer between the two organelles

**Figure 2:**
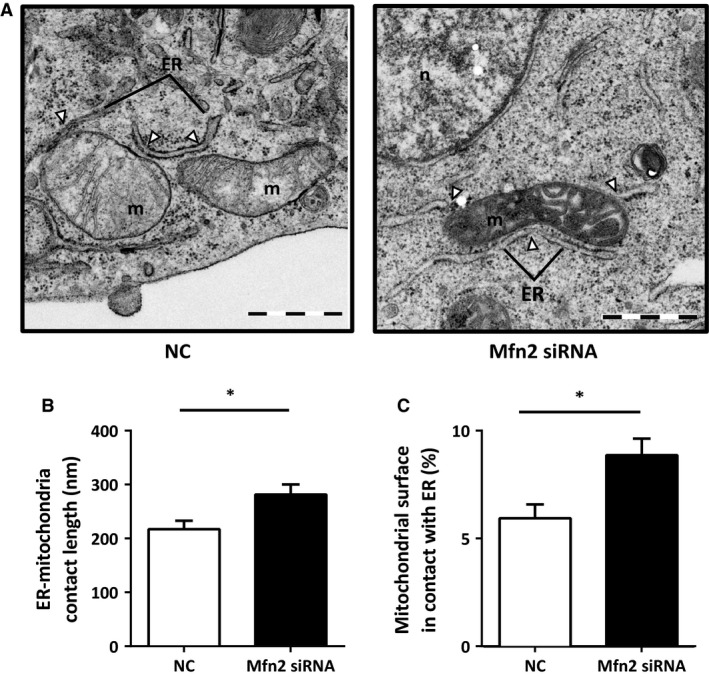
Mfn2 knockdown increases ER–mitochondria contacts. (**A**) Representative electron micrographs of HEK293 APPswe treated either with NC (left panel) or Mfn2 siRNA (right panel). Pictures were taken at a magnification of 20,500×. (**B**) Quantification of contact length in NC and Mfn2 si RNA‐treated cells. (**C**) Ratio between total length of contacts and total mitochondrial perimeter per cell. Number of cells analysed varied between 19 ≤ *n* ≤ 25. Results are shown as mean ± S.E.M. of three independent experiments. Independent *t*‐test was used for statistical analysis. *P < 0.05. NC: negative control; Mfn2 siRNA: siRNA for Mfn2 mRNA. Scale bars correspond to 1 μm.

The impact of Mfn2 knockdown on ER–mitochondria juxtaposition was analysed by transmission electron microscopy (TEM; Fig. 2A). In the analysis, ER–mitochondria contact was considered to be formed when the distance between the two membranes was ≤ 30 nm 2. Mitofusin‐2 knockdown increased the length of the ER stretches in contact with mitochondria, as compared to control (Fig. 2A and B). Accordingly, the percentage of the mitochondrial surface in contact with the ER increased in Mfn2 siRNA‐treated cells, as compared to control (Fig. 2C). The number of ER–mitochondria contacts and mitochondria, and the mitochondria perimeter, were unaltered by Mfn2 siRNA (Fig. S1A and B). Mitochondrial morphology was affected, as shown by dilation of mitochondrial cristae in Mfn2 siRNA‐treated cells (Fig. 2A).

**Figure 3:**
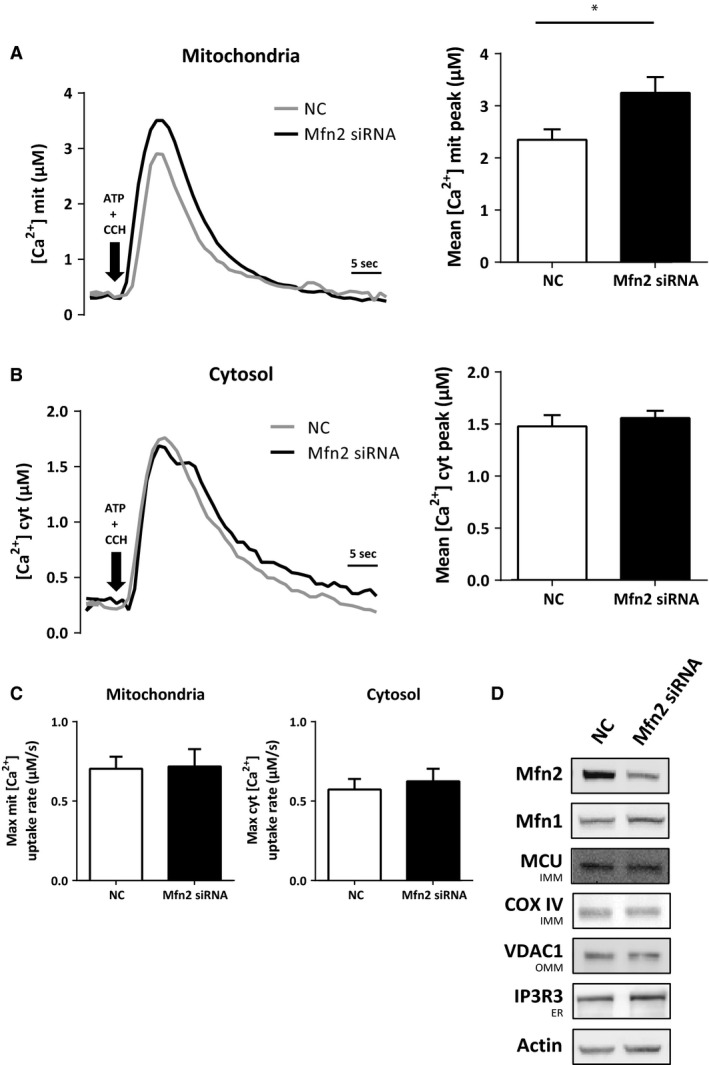
Mfn2 knockdown increases Ca^2+^ transfer between ER and mitochondria. (**A**) Mitochondrial Ca^2+^ peak upon stimulation of ER Ca^2+^ release with ATP and carbachol (CCH) or (**B**) cytosolic Ca^2+^ peak in NC and Mfn2 siRNA‐treated cells and (**C**) respective rise rates. Sample size varied between 8 ≤ *n* ≤ 10 (independent experiments), with triplicates for each condition. Results are shown in mean ± S.E.M. Independent *t*‐test was used for statistical analysis. *P < 0.05. (**D**) Western blot of whole cell homogenates from cells treated either with NC or Mfn2 siRNA. Membranes were stained for indicated proteins. COX IV was used as a mitochondria marker NC: negative control; Mfn2 siRNA: siRNA for Mfn2 mRNA.

To elucidate whether the increased physical coupling between the two organelles had a functional impact, we analysed the Ca^2+^ transfer from ER to mitochondria. An increased juxtaposition between the two organelles favours the shuttling of Ca^2+^, while a decreased juxtaposition decreases the Ca^2+^ shuttling 39, 40. To test the efficiency of Ca^2+^ transfer, HEK293 APPswe cells were cotransfected with cDNA encoding for the mitochondrial matrix‐targeted Ca^2+^ probe, aequorin, and the Mfn2 (or NC) siRNA. After 48 hrs, cells were stimulated with ATP and carbachol (CCH) to induce an IP3‐dependent ER Ca^2+^ release. A significant increase in mitochondrial Ca^2+^ peaks was observed upon Mfn2 siRNA knockdown, as compared to NC (Fig. 3A). These results show that mitochondrial Ca^2+^ uptake, upon ER Ca^2+^ release, can be modulated by the proximity of the two organelles. In addition, mitochondrial Ca^2+^ uptake may also depend on the amount of Ca^2+^ released from the ER facing mitochondria and on the levels of MCU. To investigate whether the two latter features contributed to the observed phenomenon, we first measured the cytosolic Ca^2+^ peaks obtained upon stimulation with ATP and CCH in cells expressing cytosolic aequorin (Fig. 3B). Secondly, we investigated mitochondrial Ca^2+^ uptake in permeabilized cells exposed to fixed amounts of Ca^2+^ (Fig. S2A and B). No significant differences were detected between NC‐ and Mfn2 siRNA‐treated cells using these approaches (Fig. 3B and C, Fig. S2A and B). Furthermore, the protein levels of IP3R3 (mediating Ca^2+^ release from the ER) and VDAC1 and MCU (mediating mitochondrial Ca^2+^ uptake) were not affected by Mfn2 knockdown (Fig. 3D). Cytochrome c oxidase (COX IV) was used as a mitochondrial marker. These data are in accordance with our TEM results and show that Mfn2 knockdown in HEK293 APPswe cells results in an increased ER–mitochondria contact.

### Knockdown of Mfn2 leads to decreased Aβ production *via* impairment of γ‐secretase maturation and activity

**Figure 4:**
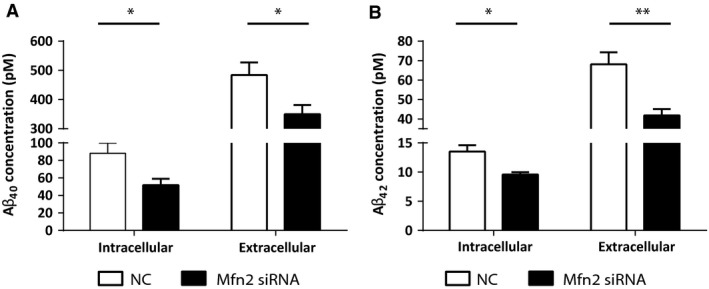
Mfn2 down‐regulation decreases intracellular and secreted levels of Aβ. Intra‐ and extracellular levels of (**A**) Aβ_40_ and (**B**) Aβ_42_ were assessed by ELISA. 4 ≤ *n* ≤ 9 (independent experiments), with duplicates for each condition. Results are shown as mean ± S.E.M. Mann–Whitney *U*‐test was used for statistical analysis. *P < 0.05, **P < 0.001. NC: negative control; Mfn2 siRNA: siRNA for Mfn2 mRNA.

Having established a cell model with increased ER–mitochondria contact, we next tested our hypothesis that the modulation of ER–mitochondria contact influences APP processing and Aβ production. Levels of intracellular and secreted Aβ were detected by a commercial ELISA. Interestingly, the levels of both intracellular and secreted Aβ_40_ and Aβ_42_ were decreased around 40% in cells treated with Mfn2 siRNA, as compared to NC siRNA (Fig. 4).

**Figure 5:**
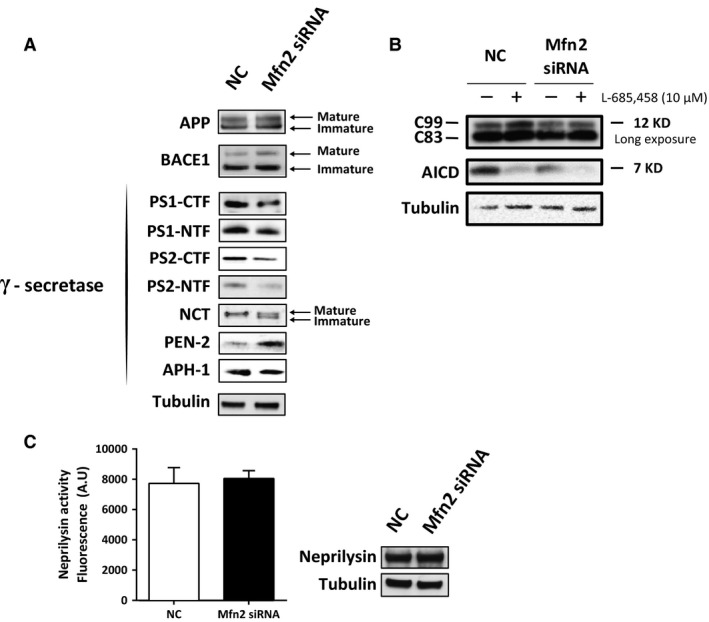
Mfn2 knockdown leads to impaired γ‐secretase activity and maturation. (**A**) Protein expression of APP, BACE1 and γ‐secretase components was assessed by Western blot analysis of cell homogenates from NC and Mfn2 siRNA‐treated cells. (**B**) γ‐secretase activity assay using a membrane fraction from cells treated with NC or Mfn2 siRNA in the presence or absence of γ‐secretase inhibitor L‐685,458. APP fragments (C99, C83, AICD) were detected using the Y188 antibody. (**C**) Intact cells were treated with NC or Mfn2 siRNA and incubated with specific neprilysin peptide. Neprilysin activity was investigated by measuring the fluorescence of cleaved peptide (arbitrary units). Western blots of cell homogenates were performed. *n* = 4 (independent experiments), with duplicates or triplicates for each condition. Results are shown in mean ± S.E.M. Mann–Whitney *U*‐test was used for statistical analysis NC: negative control; Mfn2 siRNA: siRNA for Mfn2 mRNA.

To elucidate whether the decrease in Aβ levels was as a result of impaired APP maturation, altered APP processing or increased Aβ degradation, we performed the following experiments. First, Western blot analysis revealed that the expression levels of total APP as well as of mature and immature APP were similar in Mfn2 siRNA‐ and NC siRNA‐treated cells (Fig. 5A). Amyloid‐β precursor protein is glycosylated in ER and Golgi and fully matured when reaching the plasma membrane 41. The data show that this process is not affected by Mfn2 knockdown.

Second, we detected BACE1 and γ‐secretase protein expression and measured γ‐secretase activity. Western blot studies revealed that BACE1 levels were not changed in cells treated with Mfn2 siRNA. Interestingly, levels of PS1 and PS2 C‐ and N‐terminal fragments (PS1/2‐CTFs and PS1/2‐NTFs) were significantly reduced (Fig. 5A) after Mfn2 knockdown. Moreover, levels of PEN‐2 and immature NCT increased, while APH‐1 levels were unaltered in Mfn2 siRNA‐treated cells (Fig. 5A). γ‐Secretase activity was measured in an assay detecting AICD formation from membrane fractions incubated overnight in the absence or presence of the γ‐secretase inhibitor L‐685,458. Decreased AICD production was detected in cells treated with Mfn2 siRNA as compared to NC siRNA (Fig. 5B, Fig. S3C). Levels of C83 and C99 were unaltered (Fig. 5B, Fig. S3A and B). In this assay, membrane fractions isolated from siRNA‐treated cells were incubated in a 1% CHAPSO buffer for 16 hrs. The amounts of AICD, formed from the γ‐secretase substrates C83/C99, were subsequently detected by Western blot. Our results show that sufficient amounts of C83/C99 were present in the membrane fraction, and thus decreased AICD levels reflect lower γ‐secretase activity in Mfn2 siRNA‐treated cells as compared to NC siRNA‐treated cells. Altogether these data show that increased ER–mitochondria contact negatively affects γ‐secretase complex maturation and activity.

Finally, we investigated both activity and protein levels of neprilysin. Neprilysin is a membrane‐bound zinc‐dependent metalloprotease that degrades different peptides, including Aβ. Neprilysin has been shown to be one of the major Aβ‐degrading enzymes 42. Neprilysin activity and protein expression were similar in Mfn2 siRNA‐treated cells as compared to NC siRNA‐treated cells (Fig. 5C). These data show that decreased Aβ concentrations in Mfn2 siRNA‐treated cells were not because of the increased peptide degradation by neprilysin.

## Discussion

In the recent years altered functions of MAM and ER–mitochondria communication have been described in disorders such as AD, cardiovascular disease and obesity 14, 24, 43, 44. In this study we show that Mfn2 knockdown increases ER–mitochondria juxtaposition, decreases levels of intracellular and secreted Aβ and impairs γ‐secretase activity.

The pathway for Aβ generation is well‐established, however the intracellular regulation of this process is not fully understood 29, 30, 31, 32. Several proteins have been shown to modulate γ‐secretase activity and thus Aβ production (*e.g*. VDAC1, γ‐secretase‐activating protein, reticulon protein family). These proteins present different functions and subcellular localizations within the cell 45, 46, 47, 48. Still, the exact mechanisms of how these proteins modulate γ‐secretase activity remain to be elucidated. Here, we have tested an alternative hypothesis proposing that modulation of ER–mitochondria contacts affects Aβ generation.

Mfn2 has been described to have an essential role in the tethering of ER and mitochondria 6, 7, 8, 9. As a result of its specific location and function at ER–mitochondria contacts, the ablation or reduction of this protein has been used for the modulation of ER–mitochondria contacts 6, 7, 8, 9, 15. However, the role of Mfn2 as a tethering protein between ER and mitochondria has recently been questioned by us and others 7, 8, 9. In the present study, Ca^2+^ measurements and TEM‐analysis show that the knockdown of Mfn2 results in increased coupling between ER and mitochondria. This is in accordance with recent publications proposing that Mfn2 works as an ER–mitochondria tetheering antagonist 7, 8, 9.

Mfn2 siRNA treatment also resulted in dilated mitochondrial cristae, supporting the importance of Mfn2 in mitochondrial morphology. Mitofusin‐1 and ‐2 seem to have partially redundant physiological functions, where Mfn1 can compensate for Mfn2 reduction and *vice versa* 49, 50. Accordingly, our data show that Mfn1 protein levels were slightly increased in cells treated with Mfn2 siRNA. Thus, Mfn1 could compensate for Mfn2 knockdown in terms of energy production and cell viability but not in terms of mitochondrial morphology.

In our model, the knockdown of Mfn2 siRNA and the increase in ER–mitochondria Ca^2+^ transfer did not cause mitochondrial failure and cell death (at least not within the treatment period). Ca^2+^ is crucial for cell homoeostasis. Upon certain stimuli the levels of Ca^2+^ in the cytosol can rise. Mitochondria have the ability to rapidly buffer such increased cytosolic Ca^2+^ by accumulating Ca^2+^ in the matrix. This buffering tunes overall Ca^2+^ signalling within the cell 51. Moreover, changes in mitochondrial Ca^2+^ levels can have an impact in the metabolic regulation of Ca^2+^‐activated matrix dehydrogenases, thus modulating ATP production 51, 52, 53, 54, 55. In fact, small Ca^2+^ changes within mitochondria (0.1–10 μM) can lead to major changes inside the cell 51, 52, 53, 54, 55. Disruption of Ca^2+^ homoeostasis as a result of the long or intense treatments/stimuli can lead to cell death (*e.g*. overload of mitochondrial Ca^2+^ triggers apoptosis). Several studies have shown that PS mutations lead to changes in Ca^2+^ homoeostasis by increasing cytosolic Ca^2+^ 56, 57, 58. It has also been shown that PS1 and PS2 have a γ‐secretase‐independent role in the regulation of calcium homoeostasis and tethering between ER and mitochondria 13, 56. Moreover, it has been shown that increased cytosolic Ca^2+^ leads to decreased APP levels and subsequently decreased Aβ production 59. However, in our study, Mfn2 knockdown did not result in an alteration in the bulk cytosolic Ca^2+^ concentration, neither at rest nor upon cell stimulation. Instead, the specific increase in Ca^2+^ transfer from ER to mitochondria (further demonstrating a straitened coupling between the two organelles) suggests that the observed decreased γ‐secretase activity and Aβ levels were not caused by alterations in cytosolic Ca^2+^. However, further investigations need to be done to understand the mechanism by which altered ER–mitochondria contact and Ca^2+^ transfer affect γ‐secretase maturation, assembly and activity.

Here, quantitative measurements of intra‐ and extracellular Aβ_40_ and Aβ_42_ in cells treated with Mfn2 siRNA showed that increased ER–mitochondria contact decreased Aβ levels. Possible mechanisms behind this decrease in Aβ generation include: (*i*) decreased levels or impaired maturation of APP, (*ii*) decreased expression of BACE1 and/or γ‐secretase proteins and/or activities, (*iii*) increased degradation of Aβ. Firstly, our data show that APP protein levels were unaltered and that mature APP was available for BACE1 and γ‐secretase cleavage in cells treated with Mfn2 siRNA. Secondly, BACE1 protein levels were unaltered and degradation of Aβ was not enhanced as measured by a neprilysin activity assay. Thirdly, alterations in the expression and maturation of γ‐secretase complex components were identified along with decreased γ‐secretase activity. The γ‐secretase complex assembles and matures in the secretory pathway. Nicastrin and APH‐1 first form a scaffolding complex to which PS1 (or PS2) binds. Presenilin enhancer 2 stabilizes this complex and activates endoproteolysis of PS1 (or PS2) 29, 30, 31, 32. Moreover, it has been shown that APP and active γ‐secretase complexes are enriched in lipid rafts 24, 33, 60, 61 of the plasma membrane, endosomes, autophagosomes, synaptosomes and MAM 14, 15, 16. Lipid rafts are thus important sites for Aβ production 34, 61. Here we detected both mature and immature NCT in Mfn2 siRNA‐treated cells, while cells treated with control siRNA only expressed mature NCT. APH‐1 levels were not affected by Mfn2 knockdown. The presence of immature NCT suggests that less scaffolding complexes are available for binding of PS1 (or PS2) and PEN2 under these conditions. In addition, we show that the protein levels of PS1 and PS2 (NTFs and CTFs) were decreased when Mfn2 is knocked down. γ‐Secretase complexes containing endoproteolysed PS1 are mainly responsible for the generation of Aβ, while γ‐secretase complexes containing PS2 are less active in cleaving C99 29, 30, 31, 32. PEN‐2 protein levels were increased in Mfn2 siRNA‐treated cells suggesting that PEN2 was not a limiting factor for endoproteolysis of PS. Importantly, decreased γ‐secretase activity, as measured by AICD formation, was detected. Thus, the presence of immature NCT and low levels of PS1‐NTFs and CTFs are likely the cause of low γ‐secretase activity in cells treated with Mfn2 siRNA. These results are in accordance with previous studies showing that mouse embryonic fibroblast cells lacking Mfn2 (MEF Mfn2‐KO) present a decreased activity of γ‐secretase 14.

To elucidate the exact relation between ER–mitochondria contacts and γ‐secretase activity more experiments need to be done. At present we can only speculate about the mechanisms behind the impairment of γ‐secretase complex assembly and activity. Changes in phospholipid and cholesterol metabolism and calcium homoeostasis have been linked to AD 14, 16, 62, 63. As previously mentioned, APP and active γ‐secretase complexes are enriched in lipid rafts 24, 33, 60, 61. Mitochondria‐associated ER membranes are crucial for biosynthesis of different phospholipids and cholesterol. Therefore, modulation of ER–mitochondria contact may alter MAM function and phospholipid/cholesterol synthesis resulting in altered lipid raft composition and γ‐secretase activity 14, 32, 33, 63. Furthermore, if we consider the importance of the phospholipids produced at MAM for vesicle formation, modulation of ER–mitochondria contacts could lead to altered transport/localization of γ‐secretase complexes inside the cell.

In summary, we show that γ‐secretase complex maturation and activity is affected by increased ER–mitochondria contact leading to decreased Aβ levels. More experiments are required to determine if ER–mitochondria contacts can be modulated to specifically target cleavage of APP without affecting other γ‐secretase substrates. Even though ablation of Mfn2 is not a realistic treatment strategy, because of the importance of this protein for long‐term mitochondrial stability, our data show that acute modulation of ER–mitochondria contact affects γ‐secretase assembly and function. This finding opens up for new strategies to modulate γ‐secretase activity, apart from γ‐secretase inhibitors and modulators. Altogether, the present data reinforce other studies suggesting that modulation of MAM functions and ER–mitochondria communication may hold potential as novel drug targets for future AD therapy.

## Conflict of interest

The authors confirm that there are no conflicts of interest.

## Author contribution

NSL, BS, CMP, BW and RF performed the research; NSL, BS, RF, PP and MA designed the research study; NSL, BS, CMP, RF, PP and MA analysed the data; NSL, RF, PP and MA wrote the paper.

## Supporting information


**Figure S1** Mfn2 knockdown does neither change mitochondria or contact numbers nor mitochondrial perimeter. (**A**) Quantification of the number of mitochondria and contacts in HEK293 APPswe treated either with NC or Mfn2 siRNA (**B**) quantification of mitochondrial perimeter. Quantifications were performed with Image J. Number of cells analysed varied between 19 ≤ *n* ≤ 25. Results are shown in mean ± S.E.M. of three independent experiments. Independent *t*‐test was used for statistical analysis. NC: negative control; Mfn2 siRNA: siRNA for Mfn2 mRNA.

**Figure S2** Mfn2 down‐regulation neither changes the Ca^2+^ uptake by mitochondria in permeabilized cells nor the rate of Ca^2+^ uptake. (**A**) Mitochondrial Ca^2+^ uptake in permeabilized cells exposed to specific Ca^2+^ concentrations, measured by a mitochondrial aequorin as Ca^2+^ probe. (**B**) Respective Ca^2+^ uptake rate. Sample size varied between 3 ≤ *n* ≤ 4 (independent experiments), with triplicates for each condition. Results are shown in mean ± S.E.M. Mann–Whitney *U*‐test was used for statistical analysis. NC: negative control; Mfn2 siRNA: siRNA for Mfn2 mRNA.

**Figure S3** Mfn2 knockdown leads to changes in AICD levels, while C99 or C83 levels remain unaltered. Quantification of (**A**) C83, (**B**) C99 and (**C**) AICD bands from Figure 5. Results are shown in mean ± S.E.M. 4 ≤ *n* ≤ 6. Mann–Whitney *U*‐test was used for statistical analysis. NC: negative control; Mfn2 siRNA: siRNA for Mfn2 mRNA. Membrane fractions were incubated in the presence (+) or absence (‐) of 10 μM L‐685,458 (γ‐secretase inhibitor).

**Data S1** Material and methods.
