# Supplementary material for: Mitofusin‐2 knockdown increases ER–mitochondria contact and decreases amyloid β‐peptide production
Source: J Cell Mol Med. 2016 May 20;20(9):1686–95. doi: 10.1111/jcmm.12863 (PMC4988279; doi:10.1111/jcmm.12863)
Supplement: Supplementary file 4 — Data S1 Material and methods. [file JCMM-20-1686-s004.docx]

**Supporting information**

**SI Material and Methods**

**Antibodies**

Mouse anti-Mfn2 (Abcam, #Ab56889), Rabbit anti-Mfn1 (Santa-Cruz, #SC50300), mouse anti-VDAC1 (Abcam, #Ab14734), mouse anti-TIM23 (BD Biosciences, #611223), mouse anti-Cytochrome c (BD Biosciences, #556433), mouse anti-IP3R3 (BD Biosciences, #610312), rabbit anti-APP Y188 (Abcam, #ab32136), mouse anti-PS1-CTF (Milipore, #MADB5232), rabbit anti-PS1-CTF (Calbiochem, #pc267), rabbit-PS1-NTF (Calbiochem, #529591), rabbit anti-PS2-CTF (Cell signaling, #D30G3), mouse anti-PS2-NTF (Covance, MMS-359S), mouse anti-Nicastrin (BD Bioscience, #612290), rabbit anti-APH-1aL (Biolegend, #PRB-550p), rabbit anti-PEN-2 (UD1) (kind gift from Dr Jan Näslund), rabbit anti-BACE-1 (Cell Signalling, #D10E5), rat anti-Tubulin (Abcam #ab6160), mouse anti-GAPDH (Enzo LifeScience #ADI-CSA-335-E), mouse anti-Actin (Sigma-Aldrich, #A4700), mouse anti-COX IV (Life Technologies, #459600), rabbit anti-MCU (Sigma-Aldrich, #HPA016480).

**Cell culture and transfection.**

Human Embryonic Kidney 293 (HEK293) cells stably expressing human APP with the Swedish mutation (HEK293 APPswe) (kind gift of Dr Johan Lundkvist) were cultured in DMEM supplemented with 10 % fetal bovine serum (FBS) and 100 µg/mL Zeocin. Non-transfected HEK293 cells (HEK293 wt) were cultured in the same conditions but without Zeocin. siRNA solutions were prepared by mixing Lipofectamine® RNAiMAX Transfection Reagent with either Mfn2 siRNA mix (AAGACTATAAGCTGCGAATTA, CTCTATCGTCACAGTCAAGAA, ATGGACAGCCCTGGTATTGAT, CTGCACCGCCACATAGAGGAA – SI04375406, SI04342716, SI04217430 and SI04188835 respectively, Qiagen) or AllStars Negative Control (NC) siRNA #1027280, QIAGEN) in OptiMEM to a final concentration of siRNA equal to 20 nM. The mix was incubated at room temperature (RT) for 10 minutes to allow formation of the complexes. 3-5 × 10^5^ cells were added to the mix and grown for 48h in these conditions before further treatment.

**Cell lysis and protein concentration**

siRNA treated cells were lysed in RIPA buffer [50 mM Tris (pH 7.5), 150 mM NaCl, 1 % Triton X-100, 0.5 % Deoxycholic acid, 0.1 % SDS], 1× protease inhibitor cocktail (G-Biosciences, #786-331) and benzonase solution [50 mM of Tris (pH 8.0), 4 mM MgSO_4_ and dilution 1:2000 (V/V) of Benzonase (Sigma-Aldrich). Protein concentration was determined by the Bicinchoninic Acid protein (BCA^TM^) assay.

**Western blot**

For detection of protein expression by Western blot (WB) equal amounts of protein (15-30 μg) from each sample were run on 4 %-12 % gel (Novex) and transferred to nitrocellulose membrane (GE Healthcare). Membranes were blocked in 5% milk TBS-T, incubated overnight at 4°C with first antibody, washed in TBS-T, incubated for 1 h at room temperature with secondary antibody in 5 % milk and finally washed in TBS-T. The membranes were developed using Immobilon Western Chemiluminescent HRP substract (Millipore).

**Aequorin measurements**

100.000 HEK293 APPSwe cells were seeded in coated poly-D Lysine coverslips (13 mm diameter). After 24 h, cells were transfected with Mfn2 siRNA or NC and with either cytosolic or mitochondrial aequorin cDNA for 48h. Coverslips with cells were incubated with 5 μM coelenterazine for 1 h and then placed in the perfusion chamber. Cells were perfused with Krebs–Ringer modified buffer (KRB; in mM: 135 NaCl, 5 KCl, 0.4 KH_2_PO_4_, 1 MgSO_4_, 1 MgCl_2_, 20 Hepes, 11 Glucose, pH 7.4 at 37^o^C) supplemented with 1 mM CaCl_2_ and then were stimulated with a mix of IP3-generating stimuli, 100 µM ATP and carbachol 300 µM CCH diluted in the same solution without CaCl_2_ and in the presence of 600 µM EGTA.

Experiments were terminated by lysing the cells with 100 μM digitonin in a hypotonic Ca^2+^-rich solution (10 mM CaCl_2_ in H_2_O), thus discharging the remaining aequorin pool. The light signal was collected and calibrated into Ca^2+^ concentrations as previously described [64] . In the experiments with permeabilized cells, the following intracellular-like medium was used: in mM, 130 KCl, 10 NaCl, 1 KH_2_PO_4_, 2 succinic acid, 1 MgSO_4_, 20 Hepes, 0.05 EGTA (pH 7 at 37 °C). Cells were permeabilized by 1-min perfusion with 100 μM digitonin dissolved in the intracellular-like medium. After permeabilization, digitonin was removed by 2-min washing and mitochondrial Ca^2+^ uptake was measured by mitochondria aequorin bathing cells with the same intracellular solution without EGTA and containing Ca^2+^ at different fixed concentrations (7.5, 10 and 15 μM). Mitochondrial Ca^2+^ uptake speed was calculated as the first derivative of calcium uptake by time. The higher value reached represented the maximal Ca^2+^ uptake speed. All materials were from Sigma-Aldrich unless specified differently.
